# Novel combination coccospheres from *Helicosphaera* spp indicate complex relationships between species

**DOI:** 10.1093/plankt/fbac044

**Published:** 2022-08-17

**Authors:** Daniela Sturm, Gerald Langer, Glen Wheeler

**Affiliations:** The Marine Biological Association of the United Kingdom, The Laboratory, Citadel Hill, Plymouth PL1 2PB, Devon, UK; Ocean and Earth Science, University of Southampton, University Rd, Highfield, Southampton SO14 3ZH, Hampshire, UK; Institute of Environmental Science and Technology (ICTA), Universitat Autonoma de Barcelona, Carrer de les Columnes s/n, Barcelona 08193, Spain; The Marine Biological Association of the United Kingdom, The Laboratory, Citadel Hill, Plymouth PL1 2PB, Devon, UK

**Keywords:** coccolithophores, life-cycle, combination-coccosphere, haplo-diplontic, Southern Ocean

## Abstract

Coccolithophores play an important role in global biogeochemical cycling, but many aspects of their ecology remain poorly understood, including their heteromorphic haplo-diplontic life cycle. The presence of combination coccospheres in environmental samples, which represent a transition between the lightly calcified haploid (HOL) and heavily calcified diploid (HET) life phases, provides crucial evidence linking the two life cycle phases of a particular species. Here, we describe combination coccospheres from the Southern Ocean that show a novel association between *Helicosphaera hyalina* (HET) and *Helicosphaera* HOL *catilliferus* type. The ability of *Helicosphaera* HET and HOL morphospecies to form multiple different combinations indicates a substantial complexity in the relationships between life cycle phases in this group. The findings suggest recent divergence within the *Helicosphaera* lineage may have resulted in significant inter- and intra-specific variability, with cryptic speciation in one or both life cycle phases contributing to their ability to form multiple HET/HOL associations.

## INTRODUCTION

Coccolithophores (Haptophyta) are calcified unicellular phytoplankton that play an important role in biogeochemical cycling. They exhibit a haplo-diplontic heteromorphic life cycle that remains poorly understood (Frada et al., 2019). Coccolithophores exist in either haploid (1N) or diploid (2N) life cycle phases that can each undergo asexual reproduction, although the factors that trigger a switch between life cycle phases remain unclear. Both phases are morphologically distinct, with the heavily calcified 2N phase producing the characteristic heterococcoliths (HET) and the usually lightly calcified 1N phase producing holococcoliths (HOL), which are formed from small rhombohedral crystallites (Young et al., 2003). The HET and HOL life cycle phases exhibit distinct but overlapping geographical distributions that may allow species to expand their ecological niche (De Vries et al., 2021; Frada et al., 2019).

As life cycle transitions are not commonly observed in laboratory cultures, environmental observations of combination coccospheres have provided crucial information for the study of coccolithophore life cycles. Combination coccospheres contain coccoliths from the previous and the new life-cycle phase, i.e. both heterococcoliths and holococcoliths. Critically, they have allowed researchers to link morphologically distinct cell types that were previously thought to be different taxa, but are in fact the HOL and HET phases of the same species (Triantaphyllou et al., 2015; Young et al., 2020). However, combination coccospheres have not been recorded for all species and in some cases relationships between HET and HOL phases have not been straightforward, indicating that much remains to be learnt about coccolithophore life cycles.

In this study, we examined the occurrence and identity of combination coccospheres in the diverse coccolithophore communities found within the Great Calcite Belt (GCB) of the Southern Ocean (Balch et al., 2011).

## METHODS

Water samples for coccolithophore analysis were collected from the surface mixed layer in February 2020 within a mesoscale meander of the Southern Subtropical Front (STF) in the Southern Indian Ocean (38.5–41.5°S, 30.9–35.5°E). We vacuum-filtered 150 mL of seawater, prepared samples for scanning electron microscopy (SEM) and imaged as described in Langer et al. (2021).

## RESULTS

**Fig. 1:**
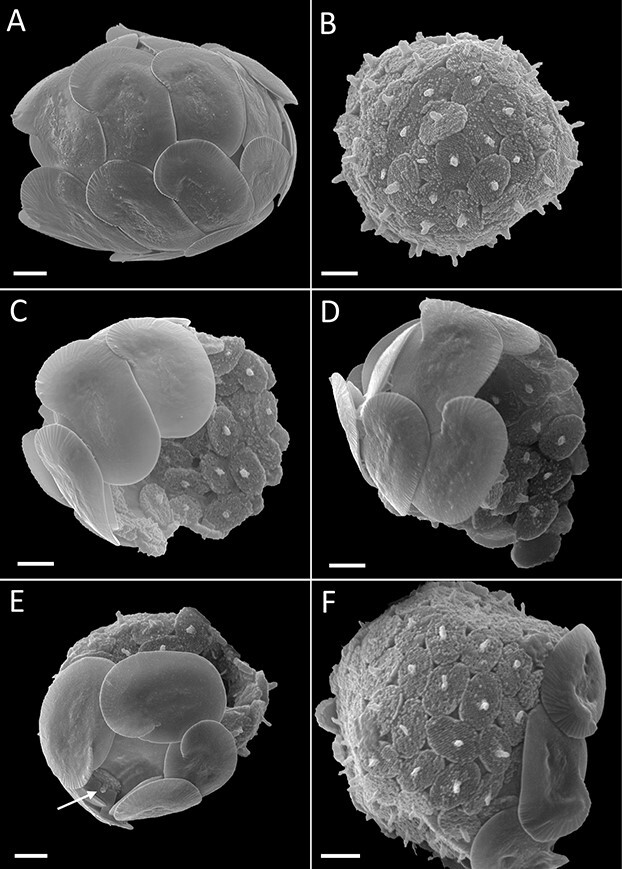
Scanning electron micrographs of *Helicosphaera* from the Southern Ocean. A: HET phase of *Helicosphaera hyalina*. Note that there is no transverse bar separating two aligned openings of heterococcoliths, as in *H. carteri*, B: HOL phase of *Helicosphaera catilliferus* type, C–F: Combination coccospheres of *H. hyalina* (HET) and *H.* HOL *catilliferus* type from the Southern Indian Ocean. Note the holococcolith protruding out from in between the heterococcoliths in image E. Scale bars 2 μm.

All identified combination coccospheres (*n* = 7) belonged to the genus *Helicosphaera* (Order Zygodiscales) and represented a novel association between *Helicosphaera hyalina* (HET) and *H.* HOL *catilliferus* type (Fig. 1C–F). The heterococcoliths possessed well-developed wings in the distal flange and a closed central area (Fig. 1A). Holococcoliths were elliptical with a sharply pointed central protrusion (Cros and Fortuño, 2002) (Fig. 1B). All combination coccospheres were nearly perfectly preserved, suggesting that they do not represent a sampling artifact e.g. from collisions between cells.

## DISCUSSION

**Fig. 2:**
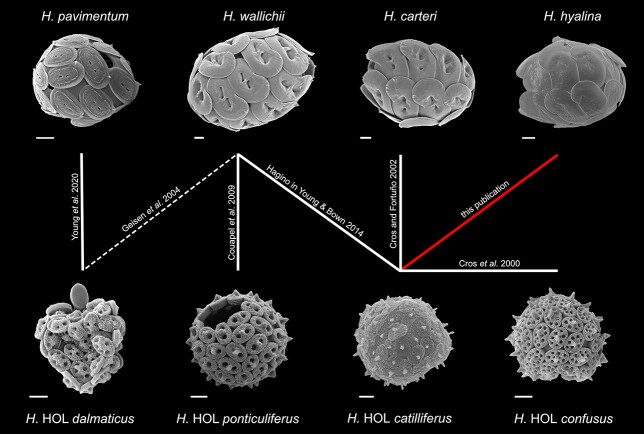
Schematic showing the currently known combination coccospheres of the group *Helicosphaera* spp based on the HOL morphotype nomenclature established by Young and Bown (2014). SEM images of HET *H. pavimentum, wallichii, hyalina* and HOL types *dalmaticus, ponticuliferus* and *confusus* were used from Nannotax3 with permission. Scale bars 2 μm.

*Helicosphaera* are medium to large species with heavily calcified heterococcoliths and a global distribution (Šupraha and Henderiks, 2020). There is continued uncertainty about the relationships between HET and HOL morphotypes within *Helicosphaera,* as multiple combination coccospheres have been described and there does not appear to be a simple 1:1 correlation of HET and HOL types, as in most other coccolithophore groups (Fig. 2). *H. wallichii* (HET) has been associated with the HOL types *ponticuliferus* and *catilliferus* (Couapel et al., 2009; Young and Bown, 2014). The weak evidence for an additional interaction with HOL type *dalmaticus* probably represents an accidental association (Geisen et al., 2004; Young and Geisen, 2002). The HOL type *catilliferus* has in addition been found in combination with HET coccoliths of *Helicosphaera carteri,* and the HOL type *dalmaticus* is also associated with *H. pavimentum* (HET) (Cros et al., 2000; Young et al., 2020). Our finding adds the further combination of *H. hyalina* (HET) and HOL type *catilliferus.*

These observations render the question whether (1) these *Helicosphaera* morphotypes represent a single species where genetic variability leads to the formation of distinct coccolith morphologies, or (2) different *Helicosphaera* morphotypes represent diverging species that have not yet lost (or have regained) the ability to hybridize (introgressive hybridization), enabling the formation of multiple combination coccospheres. For clarity, we have used the term morphotypes below to differentiate between cell types, although their exact relationships remain unclear.

*H. hyalina* HET and *H. wallichii* HET were initially regarded as varieties of *H. carteri* HET due to their similar morphology (Cros and Fortuño, 2002; Jordan and Green, 1994). Analysis of fossil *Helicosphaera* populations over the last 15 Myr indicates an evolutionary trend favoring smaller celled species (like *H. hyalina*) that may have been driven by the prevalence of oligotrophic conditions and could have contributed to divergence within this lineage (Šupraha and Henderiks, 2020).

Molecular evidence is now needed to unveil whether these *Helicosphaera* morphotypes represent distinct species, and if they do, when and how they emerged. Sequencing of the fast-evolving chloroplast gene tufA showed multiple substitutions between *H. carteri* and *H. hyalina,* suggesting a recent pseudocryptic speciation event (Sáez et al., 2003). However, there is currently no genetic information for any other *Helicosphaera* HET or HOL morphotypes that would allow a comparison with *H. carteri.* Our newly described association between *H. hyalina* and *H.* HOL *catilliferus* indicates that *H. hyalina* does in fact belong to the *H. wallichii–carteri* species complex. Distinct distributions (De Vries et al., 2021) within this *wallichii–carteri–hyalina* group suggest that the variability within this super-species allows the expansion of their geographical niche. The question of how phytoplankton species originate and evolve remains elusive. The processes required for genetic differentiation of terrestrial organisms, i.e. limited gene flow through dispersal barriers, natural selection, mutation or genetic drift are not always clear in the open ocean (Rengefors et al., 2017). Despite this, eukaryotic phytoplankton are hugely diverse in their phylogeny, physiology and morphology (De Vargas et al., 2015). Recent whole-genome analysis from another coccolithophore group, *Gephyrocapsa/Emiliania*, suggests that speciation within this group occurred during a period of physical isolation, and was followed by recent secondary contact leading to interspecific hybridization and gene flow (Filatov et al., 2021). A similar situation of divergence followed by secondary contact within *Helicosphaera* could provide an explanation for the multiple associations between HET/HOL morphotypes. Evidence of associations of the HOL types *catilliferus* and *confusus* could represent an indication of sexual reproduction, i.e. hybridization between distinct *Helicosphaera* species (Cros et al., 2000).

Note that combination coccospheres are not observed in *Gephyrocapsa/Emiliania* as the haploid phase is not calcified (Frada et al., 2019).

## CONCLUSION

Our observation provides additional evidence for a complex relationship between HET and HOL morphotypes within *Helicosphaera*, highlighting the need to better understand coccolithophore life cycles and the processes of speciation within phytoplankton. In addition to the morphological analysis of the fossil record, the development of techniques to link genetic information to morphological phenotypes, e.g. through targeted single-cell genomic sequencing of environmental samples, may enable us to address these questions in the near future.
